# Rapid and Lasting Effects of Activating BDNF-Expressing PVH Neurons on Energy Balance

**DOI:** 10.1523/ENEURO.0009-22.2022

**Published:** 2022-04-05

**Authors:** Shaw-wen Wu, Baoji Xu

**Affiliations:** Department of Neuroscience, the Scripps Research Institute, Jupiter, FL 33458

**Keywords:** BDNF, energy expenditure, food intake, neuronal activation, paraventricular hypothalamus, respiratory exchange ratio

## Abstract

Brain-derived neurotrophic factor (BDNF) and its receptor, tropomyosin receptor kinase B (TrkB), are implicit in causing obesity. Mutations that reduce BDNF and TrkB expression are associated with obesity in humans and mice. Recently, it was reported that *Bdnf* gene deletion in the neurons of the paraventricular hypothalamus (PVH) caused positive energy balance and severe obesity in the form of hyperphagia, impaired adaptive thermogenesis, and decreased energy expenditure. Thus, we hypothesize that activation of these neurons will have the opposite effect and provide an opportunity for long-lasting obesity treatment. To specifically activate BDNF-expressing PVH (PVH^BDNF^) neurons, we injected Cre-dependent adeno-associated virus (AAV) expressing the excitatory DREADD hM3Dq bilaterally into the PVH of *Bdnf^2A-Cre/+^* knock-in mice and then administered clozapine-N-oxide (CNO). Using this technique, we demonstrated that acute activation of these neurons rapidly decreased normal nocturnal feeding and fasting-induced feeding in male and female mice. At thermoneutral temperatures, acute activation also rapidly increased adaptive thermogenesis, increased core body temperature, increased locomotion, increased energy expenditure, and decreased respiratory exchange ratio (RER) in male and female mice. These observations indicate that acute stimulation of PVH^BDNF^ neurons promotes negative energy balance and weight loss. However, the rapid decrease in RER after activation of PVH^BDNF^ neurons was followed by a delayed and prolonged increase in RER that remained elevated for 3 d in female mice. Thus, although acute activation of PVH^BDNF^ neurons promotes negative energy balance in the short term, long-term effects of activation include sexually dimorphic overcompensatory mechanisms that may promote positive energy balance in female mice.

## Significance Statement

Brain-derived neurotrophic factor (BDNF) expression in the paraventricular hypothalamus (PVH) is critical for control of energy balance but it remains to be shown whether BDNF-expressing PVH (PVH^BDNF^) neurons are actively involved in control of energy balance. To answer this question, we used a chemogenetic approach to directly stimulate these neurons and measured parameters of energy balance. This study shows that acute PVH^BDNF^ neuronal stimulation initially shifts the biological system toward negative energy balance, but in females this is followed by a compensatory shift toward decreased fatty acid oxidation, leading to increased adiposity in the long-term. We speculate that this overcompensation in lipid metabolism could be a mechanism for sexual dimorphism in obesity, which is more severe in women than in men.

## Introduction

Obesity is a rising epidemic associated with disorders such as diabetes, heart disease, and cancer (Calle and Kaaks, 2004; Calle and Thun, 2004; Poirier et al., 2006; Ng et al., 2014; Ogden et al., 2014a,b). Over two-thirds of adults in the United States are overweight and over one-third are obese (Ogden et al., 2014a,b). Increased body weight is caused by a chronic energy imbalance because of increased food intake and/or decreased energy expenditure (Spiegelman and Flier, 2001; Berthoud and Morrison, 2008). In addition, there are few therapeutic options available for long-lasting treatment of obesity.

Human genome wide association studies have identified a select number of obesity-associated genes (Thorleifsson et al., 2009; Speliotes et al., 2010). Of these genes, brain-derived neurotrophic factor (BDNF), a growth factor essential for neuronal development and synaptic function, contributes to obesity (Rios, 2013; Xu and Xie, 2016). Mutations in BDNF and its receptor, tropomyosin receptor kinase B (TrkB), are associated with hyperphagia and obesity in mice and humans (Rios et al., 2001; Xu et al., 2003; Yeo et al., 2004; Gray et al., 2006; Han et al., 2008). It was recently discovered that BDNF expression in the paraventricular hypothalamus (PVH) is critical for the control of energy balance (An et al., 2015). In addition, site-specific deletion of the *Bdnf* gene in the adult PVH increases food intake, decreases physical activity, and interferes with adaptive thermogenesis in brown adipose tissues (BATs), consequently resulting in severe obesity in mice (An et al., 2015). However, it remains unknown whether BDNF-expressing PVH (PVH^BDNF^) neurons are actively involved in the control of energy balance and whether activation of PVH^BDNF^ neurons presents an opportunity for obesity treatment.

To determine the role of PVH^BDNF^ neurons in the control of energy balance, we used a chemogenetic approach to directly stimulate these neurons and measured parameters of energy balance. We found that acute stimulation of PVH^BDNF^ neurons causes a decrease in food intake (both nocturnal and fasting induced). It also causes rapid increases in adaptive thermogenesis, physical activity, core body temperature, energy expenditure, and oxidation of fatty acids. However, a rapid increase in fatty acid consumption is followed by a long-lasting decrease in fatty acid consumption after PVH^BDNF^ neuronal stimulation specifically in females. Lastly, chronic PVH^BDNF^ neuronal stimulation in female mice does not change body weight but causes an increase in fat mass and a decrease in lean mass.

## Materials and Methods

### Animal studies

The *Bdnf^2A-Cre^* knock-in mouse strain (Tan et al., 2016) was obtained from the Jackson Laboratory (stock #030189). Homozygous *Bdnf^2A-Cre^* mice were crossed with C57BL/6J wild-type mice to yield *Bdnf^2A-Cre/+^* male and female mice which were used for all experiments. Mice were kept in a 22°C room and maintained on a 12/12 h light/dark cycle with *ad libitum* access to water and regular rodent chow (Teklad Rodent Diet 2920). All animal studies were conducted in accordance with animal procedures approved by the institutional animal care and use committee at the Scripps Research Institute.

### Stereotaxic injection of adeno-associated virus (AAV)

Mice were deeply anesthetized with isoflurane before being fixed onto a stereotaxic frame. A small incision was made on the skull of each mouse to expose the brain and a small hole was drilled on the skull to allow for injection. Afterwards, stereotaxic viral injections were performed using a 2.5-μl Hamilton syringe and 33-gauge needle (World Precision Instruments) attached to a microsyringe nanopump (World Precision Instruments, UMP3 pump and Micro4 controller) and digital stereotaxic apparatus (David Kopf Instruments). AAV2-hSyn-DIO-hM3D(Gq)-mCherry or AAV2-hSyn-DIO-mCherry (packaged by Vigene Biosciences) was bilaterally injected into the PVH of 8- to 10-week-old *Bdnf^2A-Cre/+^* mice. The following coordinates (anteroposterior relative to bregma: −0.6 mm; mediolateral: ±0.3 mm; dorsoventral: −5 to −5.44 mm) were used to inject 100 nl of virus at an infusion rate of 20 nl/min using a micropump and controller (World Precision Instruments, UMP3 pump and Micro4 controller). The needle remained in the PVH for 10 min to prevent backflow of the virus and infection of the injection tract. After the surgery, animals received meloxicam (5 mg/kg) for analgesia and were placed on a heat pad on medium warmth heat until they were ambulatory. Mice were monitored for 3 d after surgery. After completion of all physiological measurements, mice were killed, and brains were collected in 4% paraformaldehyde. Brains were sliced on a microtome at 40 μm and then analyzed for expression of mCherry at the injection site. Mice with missed injections were excluded in data analyses. A total of 61 male mice and 59 female mice were injected in the study, among which 27 males and 17 females were excluded.

### Physiologic measurements

Body composition was measured using a MinispecLF-50/mq 7.5 NMR Analyzer (Brucker Optics). A comprehensive lab animal monitoring system (CLAMS; Columbus Instruments) was used to obtain measurements of locomotor activity, oxygen consumption rate (VO_2_), and CO_2_ production rate (VCO_2_) at thermoneutrality (30°C) as described previously (Houtz et al., 2021). Mice were first acclimated to metabolic chambers for at least 24 h. Afterwards, an additional 24 h was used for baseline data collection of locomotor activity, VO_2_, and VCO_2_. To measure the outcome of PVH^BDNF^ neuronal stimulation, mice were intraperitoneally injected with saline at 9 A.M. and then clozapine-N-oxide (CNO; 1 mg/kg, Cayman Chemical) at 1 P.M. on the same day. Saline was used as a solvent to dissolve CNO. Thus, saline is referred to as vehicle (VEH) in the results section and the figures. Nocturnal and fasting-induced food intake measurements were conducted as described previously (Liao et al., 2019) using regular chow (Teklad Rodent Diet 2920×; 3.1 kcal/g energy density). Mice were extensively handled for 3 d and subjected to daily mock IP injections before food intake measurements. For nocturnal food intake measurements, mice were individually housed, and food was removed 3 h before lights out. 30 min before lights out, mice were injected with saline or CNO, and 10 min before lights out, food was put in and measured each hour for 4 h. For fasting-induced feeding, food intake measurements were taken during the light cycle. Food was removed right before lights out at 7 P.M. of the night before to subject the mice to 14 h of food deprivation. The light phase started at 7 A.M. the next day. At 8:30 A.M., we administered saline or CNO and then added food 20 min after. Food consumption was measured each hour for 3 h after food in during the light cycle.

### Core body temperature and thermal imaging

Mice were acclimated to thermoneutral temperature for one week before core body temperature measurements. Core body temperature was determined using a digital thermometer (Fisher Traceable type K thermometer) attached to a rectal temperature probe (World Precision Instruments, RET-3). The rectal probe was inserted 2 cm into the rectum of mice for consistent temperature readings. For thermal imaging, the back of the mice was shaved near the interscapular area to expose the interscapular BAT (iBAT). After shaving, mice were acclimated for 24 h before thermal imaging. A thermal camera was used (FLIR E53sc) to capture iBAT temperature. First, we measured temperature at baseline and then administered saline or CNO. After saline or CNO injection, temperature was captured 30 and 60 min after administration. iBAT temperature measurement after vehicle or CNO administration were measured on different days.

### Statistical analysis

All results are reported as mean values ± SEM and analyzed using GraphPad Prism software. For all comparisons, we used RM two-way ANOVA followed by a Bonferroni’s *post hoc* test or Student’s *t* test; *p* < 0.05 were considered significant.

## Results

### Stimulation of PVH^BDNF^ neurons reduces food intake

**Figure 1.**
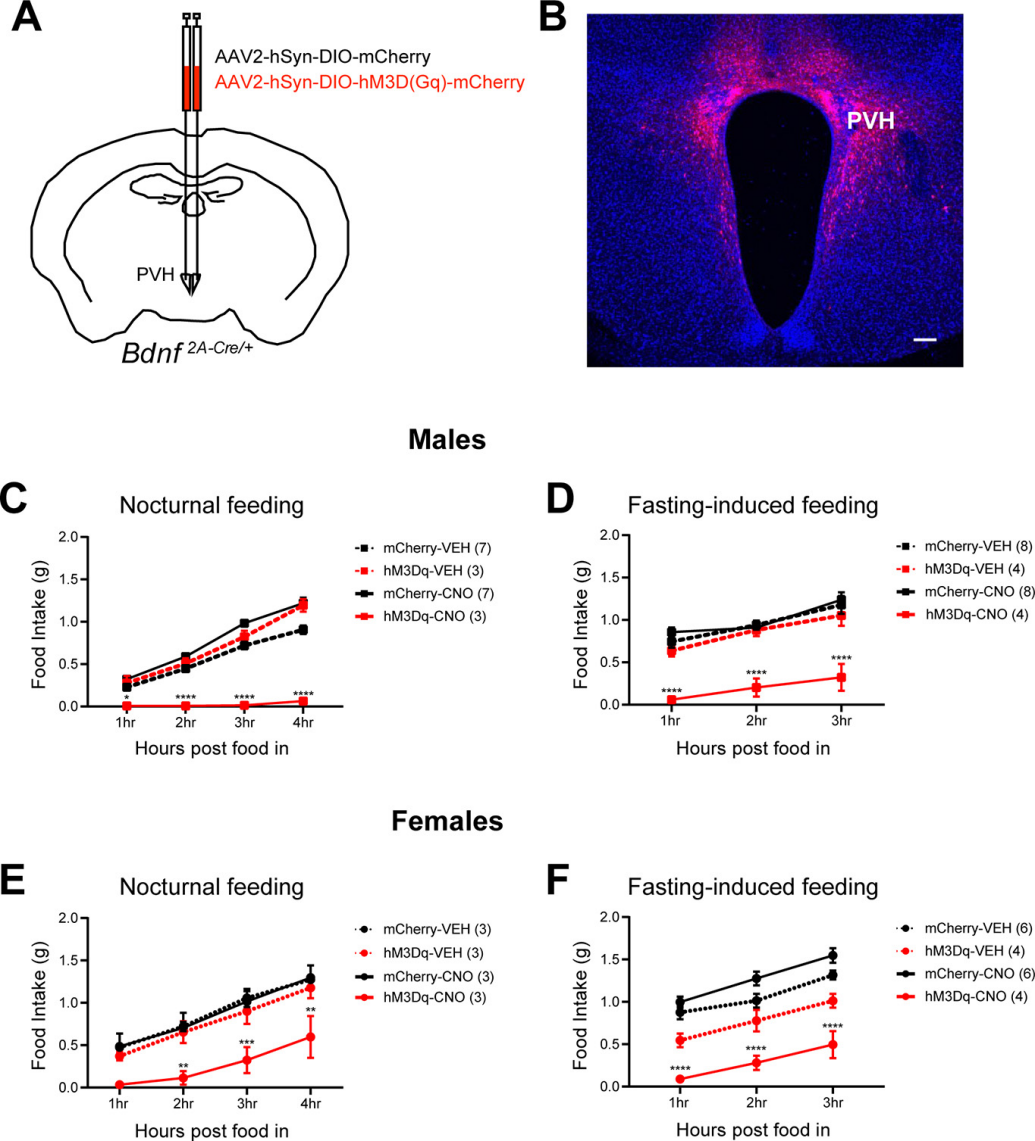
Activation of PVH^BDNF^ neurons suppresses food intake. ***A***, Illustration of bilateral injection of AAV2-hSyn-DIO-mCherry (mCherry) or AAV2-hSyn-DIO-hM3D(Gq)-mCherry (hM3Dq) into the PVH of a *Bdnf^2A-Cre/+^* mouse. ***B***, Representative image showing mCherry expression in the PVH of a *Bdnf^2A-Cre/+^* mouse. The section was counter-stained with DAPI. Scale bar: 100 μm. ***C***, Chemogenetic activation of PVH^BDNF^ neurons suppresses nocturnal food intake in male mice. Mice expressing mCherry or hM3Dq were treated with vehicle (VEH) or CNO. ***D***, Chemogenetic activation of PVH^BDNF^ neurons suppresses fasting-induced feeding in male mice. ***E***, Chemogenetic activation of PVH^BDNF^ neurons suppresses nocturnal food intake in female mice. ***F***, Chemogenetic activation of PVH^BDNF^ neurons suppresses fasting-induced feeding in female mice. Data are expressed as mean ± SEM. Animal number in each group is listed in legends. RM two-way ANOVA Bonferroni’s multiple comparisons test versus mCherry-VEH group: *p < 0.05, **p < 0.01, ***p < 0.001, and ****p < 0.0001.

To selectively activate PVH^BDNF^ neurons, we used a Cre-dependent AAV vector that expresses the excitatory designer receptor exclusively activated by designer drug (DREADD) hM3Dq (AAV2-hSyn-DIO-hM3D(Gq)-mCherry). As a control, we used a Cre-dependent AAV that only expresses mCherry (AAV2-hSyn-DIO-mCherry). These viruses were injected bilaterally into the PVH of *Bdnf^2A-Cre/+^* mice (Fig. 1*A*,*B*). As both viruses are Cre-dependent, only PVH^BDNF^ neurons will express hM3D(Gq)-mCherry (hM3Dq) or mCherry. To activate PVH^BDNF^ neurons in *Bdnf^2A-Cre/+^* mice, we administered CNO into the intraperitoneal space. CNO is a synthetic inert ligand that only targets hM3Dq such that PVH^BDNF^ neurons were activated in hM3Dq virus-infected *Bdnf^2A-Cre/+^* mice, but not in control virus-infected *Bdnf^2A-Cre/+^* mice. By using this method, we were able to chemogenetically activate PVH^BDNF^ neurons (Extended Data Fig. 1-1) to determine the acute effect on food intake. First, we looked at the effect of PVH^BDNF^ activation on nocturnal feeding. CNO administration suppressed food intake in hM3Dq-expressing male and female mice compared with vehicle administration. mCherry control mice showed no difference in food intake in response to vehicle or CNO administration (Fig. 1*C*,*E*). To measure the effect of PVH^BDNF^ neuronal stimulation on fasting-induced feeding, we fasted mice overnight and administered vehicle or CNO in the morning. Like nocturnal feeding, CNO administration also suppressed fasting-induced food intake compared with vehicle in hM3Dq-expressing mice whereas mCherry-expressing control mice showed no difference in response to vehicle or CNO administration in male and female mice (Fig. 1*D*,*F*). While there are visible qualitative differences between the female control groups (mCherry-VEH, mCherry-CNO, and hM3-VEH) in the fasting-induced feeding protocol, these differences are not statistically significant. Collectively, these results indicate that activation of PVH^BDNF^ neurons actively suppresses appetite in male and female mice.

10.1523/ENEURO.0009-22.2022.f1-1 Extended Data Figure 1-1 CNO administration induces Fos expression in hM3Dq mice. Representative images of *Bdnf^2A-Cre/+^* mice injected into the PVH with AAV2-hSyn-DIO-mCherry or AAV2-hSyn-DIO-hM3D(Gq)-mCherry (hM3Dq). Mice were treated with vehicle (VEH) or CNO (1 mg/kg) 1 h prior to euthanization. Top row, Mouse injected with AAV2-hSyn-DIO-hM3D(Gq)-mCherry (hM3Dq) and treated with VEH. Middle row, Mouse injected with AAV2-hSyn-DIO-hM3D(Gq)-mCherry (hM3Dq) and treated with CNO. Bottom row, Mouse injected with AAV2-hSyn-DIO-mCherry (mCherry) and treated with CNO. Scale bar: 100 μm. Download Figure 1-1, TIF file.

### PVH^BDNF^ neuronal stimulation increases thermogenesis and core body temperature

**Figure 2.**
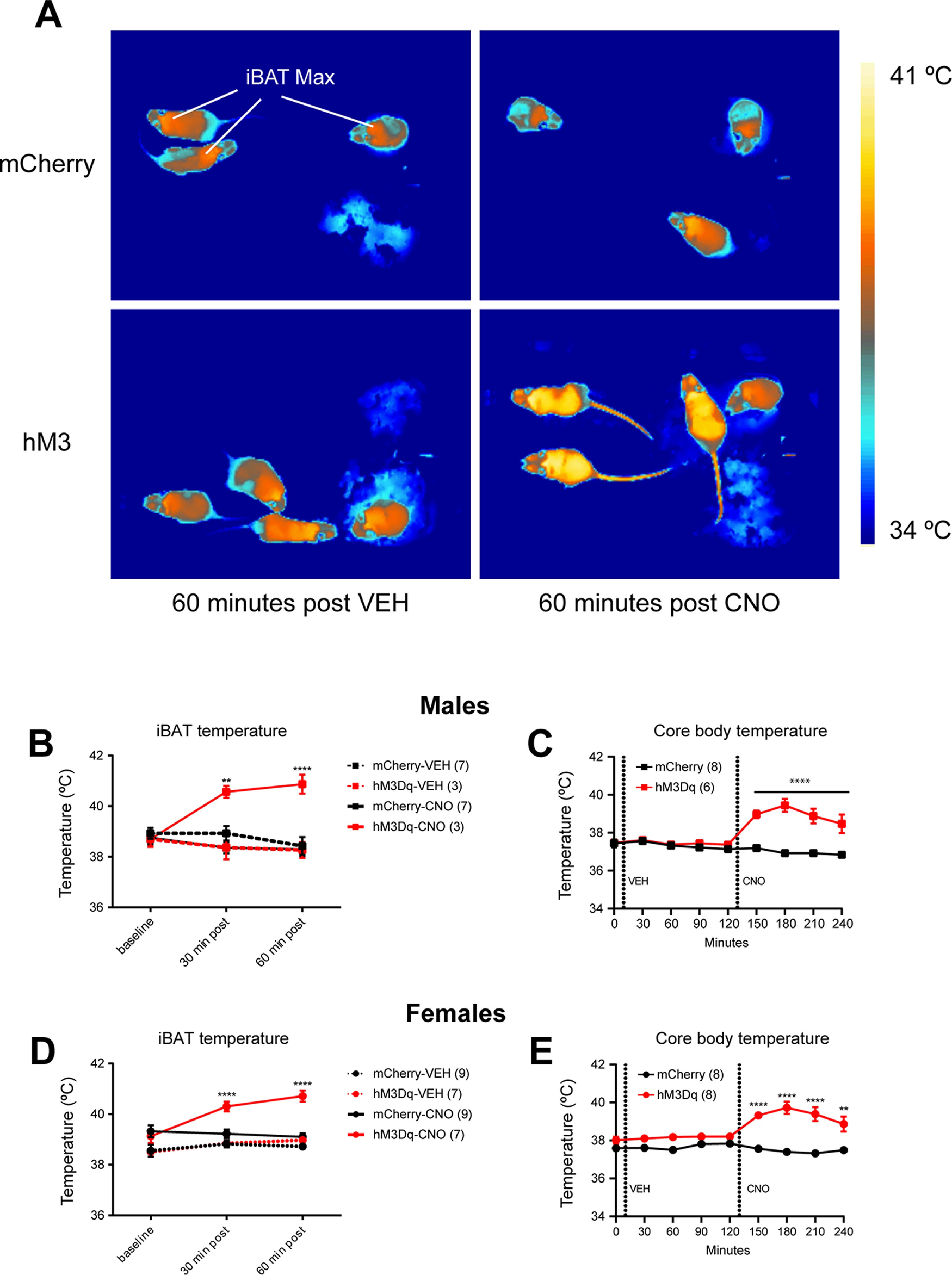
Acute PVH^BDNF^ neuronal stimulation increases thermogenesis and core body temperature. ***A***, Representative thermal images of *Bdnf^2A-Cre/+^* mice 60 min after vehicle (VEH) or CNO injection. Either AAV2-hSyn-DIO-mCherry (mCherry) or AAV2-hSyn-DIO-hM3D(Gq)-mCherry (hM3Dq) was injected bilaterally into the PVH. Lines point to areas of iBAT used to measure temperature. ***B***, iBAT (max) temperature over the course of 60 min after VEH or CNO injection in males. ***C***, Core body temperature over 4 h after sequential injection with VEH (0–2 h) and CNO (2–4 h) in male mice. ***D***, iBAT (max) temperature over the course of 60 min after VEH or CNO injection in females. ***E***, Core body temperature over 4 h after sequential injection with VEH (0–2 h) and CNO (2–4 h) in female mice. Data are expressed as mean ± SEM. Animal number in each group is listed in legends. RM two-way ANOVA Bonferroni’s multiple comparisons test versus the mCherry-VEH group (***B***, ***D***) or the mCherry group at the same time point (***C***, ***E***): **p < 0.01 and ****p < 0.0001.

As some PVH^BDNF^ neurons are polysynaptically linked to the iBAT (An et al., 2015), we hypothesize that stimulation of PVH^BDNF^ neurons will elevate iBAT adaptive thermogenesis and body temperature. We monitored adaptive thermogenesis by measuring the temperature of the skin above the iBAT (iBAT temperature) with a thermal imaging camera (Fig. 2*A*) and core body temperature with a rectal probe. CNO administration rapidly elevated iBAT temperature (Fig. 2*B*,*D*) and core body temperature (Fig. 2*C*,*E*) in *Bdnf^2A-Cre/+^* mice of both sexes expressing hM3Dq, but not mCherry, in PVH^BDNF^ neurons compared with vehicle administration. These results indicate that activation of PVH^BDNF^ neurons actively regulate iBAT adaptive thermogenesis.

### PVH^BDNF^ neuronal stimulation increases physical activity and energy expenditure

The above observation on iBAT adaptive thermogenesis suggests that PVH^BDNF^ neuronal stimulation should increase energy expenditure. As iBAT thermogenesis uses fatty acids as fuel (Harms and Seale, 2013), PVH^BDNF^ neuronal stimulation is also expected to reduce respiratory exchange ratio (RER; a ratio of VCO_2_ over VO_2_). Moreover, because deletion of the *Bdnf* gene in the PVH appears to reduce physical activity (An et al., 2015), activation of PVH^BDNF^ neurons could increase energy expenditure by promoting physical activity. To this end, we monitored the impact of vehicle or CNO administration on locomotor activity, VO_2_, and RER in *Bdnf^2A-Cre/+^* mice injected with AAV to express mCherry (control) or hM3Dq in PVH^BDNF^ neurons using CLAMS.

**Figure 3.**
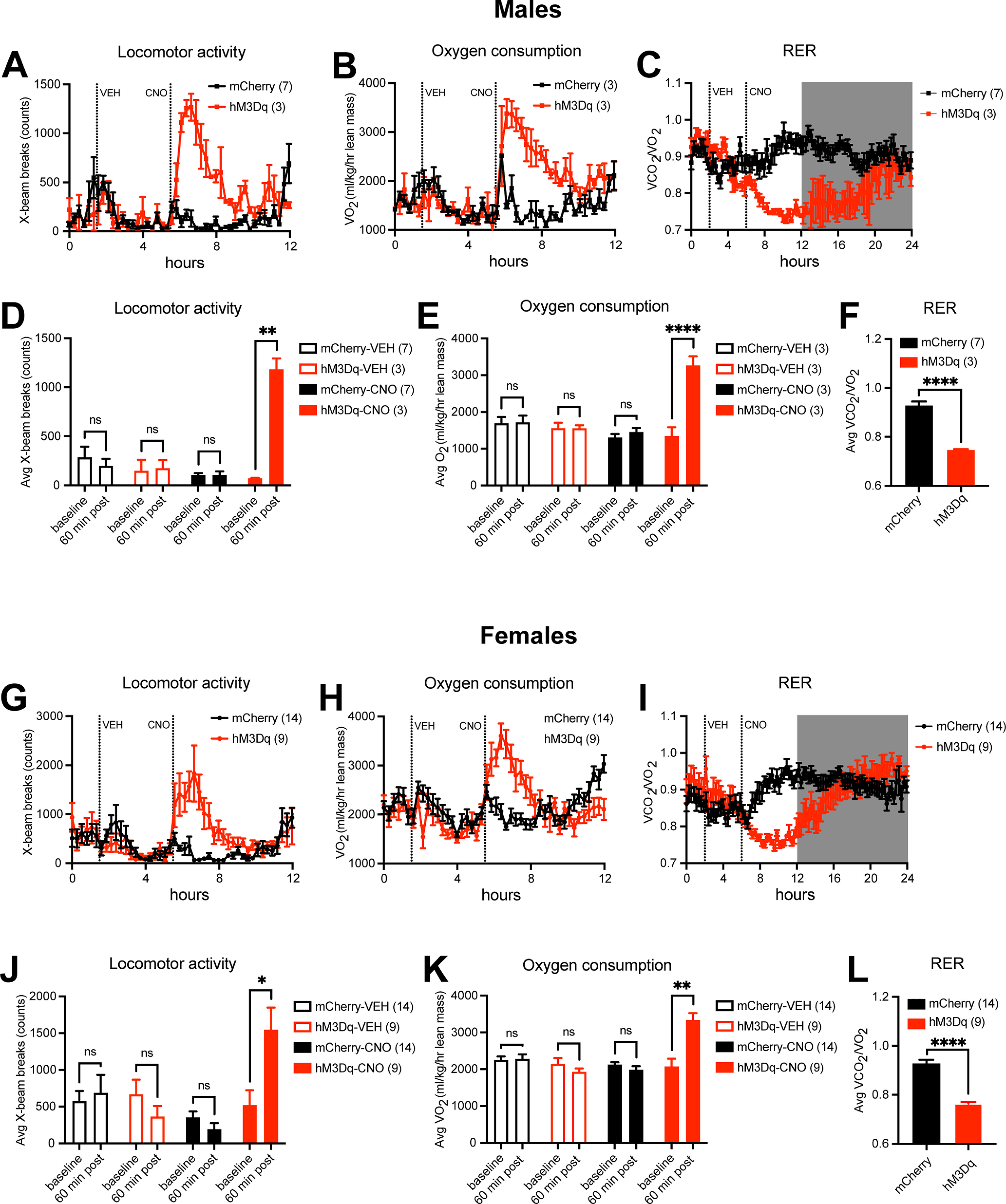
Activation of PVH^BDNF^ neurons increases energy expenditure. ***A***, ***G***, Locomotor activity over 12 h after sequential VEH and CNO injection in male and female *Bdnf^2A-Cre/+^* mice injected with either AAV2-hSyn-DIO-mCherry (mCherry) or AAV2-hSyn-DIO-hM3D(Gq)-mCherry (hM3Dq) into the PVH. RM Two-way ANOVA between mCherry and hM3Dq groups: males (over 4 h post-VEH: *F*_(1,8)_ = 0.1209 and *p* = 0.7371; over 4 h post-CNO: *F*_(1,8)_ = 119.2 and *p* < 0.0001) and females (over 4 h post-VEH: *F*_(1,21)_ = 0.3294 and *p* = 0.5721; over 4 h post-CNO: *F*_(1,21)_ = 31.50 and *p* < 0.0001). ***B***, ***H***, Oxygen consumption (VO_2_) over 12 h after sequential VEH and CNO injection in male or female mCherry or hM3Dq mice. RM two-way ANOVA between mCherry and hM3Dq groups: males (over 4 h post-VEH: *F*_(1,4)_ = 0.6077 and *p* = 0.4792; over 4 h post-CNO: *F*_(1,4)_ = 16.40 and *p* = 0.0155) and females (over 4 h post-VEH: *F*_(1,21)_ = 5.246 and *p* = 0.0238; over 4 h post-CNO: *F*_(1,21)_ = 25.11 and *p* < 0.0001). ***C***, ***I***, RER over 24 h after sequential VEH and CNO injection in male or female mCherry or hM3Dq mice. RM two-way ANOVA between mCherry and hM3Dq groups: males post-CNO: *F*_(1,8)_ = 22.04 and *p* = 0.0016; females post-CNO: *F*_(1,21)_ = 5.594 and *p* = 0.0277. ***D***, ***J***, Locomotor activity average during 1-h period before and after VEH or CNO injection in male or female mCherry or hM3Dq mice. Paired *t* test: ns = not significant, *p < 0.05, and **p < 0.01. ***E***, ***K***, Oxygen consumption average during 1-h period before and after VEH or CNO injection in male or female mCherry or hM3Dq mice. Paired *t* test: ns = not significant, **p < 0.01, and ****p < 0.0001. ***F***, ***L***, RER average during hours 8–12 in male or female mCherry or hM3Dq mice. Unpaired *t* test: ****p < 0.0001. Data are expressed as mean ± SEM. Animal number in each group is listed in legends.

We injected vehicle at 9 A.M. and saw that there was no difference in locomotion between control mice and hM3Dq mice of both sexes (Fig. 3*A*,*G*). However, injection of CNO resulted in a rapid and significant increase in locomotor activity in male and female hM3Dq mice but not control mice (Fig. 3*A*,*D*,*G*,*J*). VO_2_ is an indirect calorimetry measure for energy expenditure (Fig. 3*B*,*H*). After vehicle injection at 9 A.M., there was no difference between hM3Dq mice and mCherry control mice. After CNO injection, we observed a rapid and significant increase in oxygen consumption in hM3Dq animals but not control animals (Fig. 3*B*,*E*,*H*,*K*). Thus, PVH^BDNF^ neurons actively regulate physical activity and energy expenditure, and their activation greatly increases locomotion and oxygen consumption.

Vehicle injection showed no difference in RER between 2 groups of mice, but CNO injection caused a significant decrease in RER in hM3Dq mice compared with control mice (Fig. 3*C*,*F*,*I*,*L*). This indicates that CNO causes increases in lipolysis and β-oxidation of fatty acids. Interestingly, CNO administration qualitatively appears to result in much longer effects on RER (∼14 h in males and ∼10 h in females; Fig. 3*C*,*I*) than on locomotor activity and oxygen consumption (∼4 h; Fig. 3*A*,*B*,*G*,*H*). This discrepancy in duration of efficacy suggests that RER reduction is not a simple consequence of increased energy expenditure. Some PVH^BDNF^ neurons may directly regulate relative utilization of carbohydrates versus fatty acids as fuel.

### Activation of PVH^BDNF^ neurons leads to sustained increase in female RER

**Figure 4.**
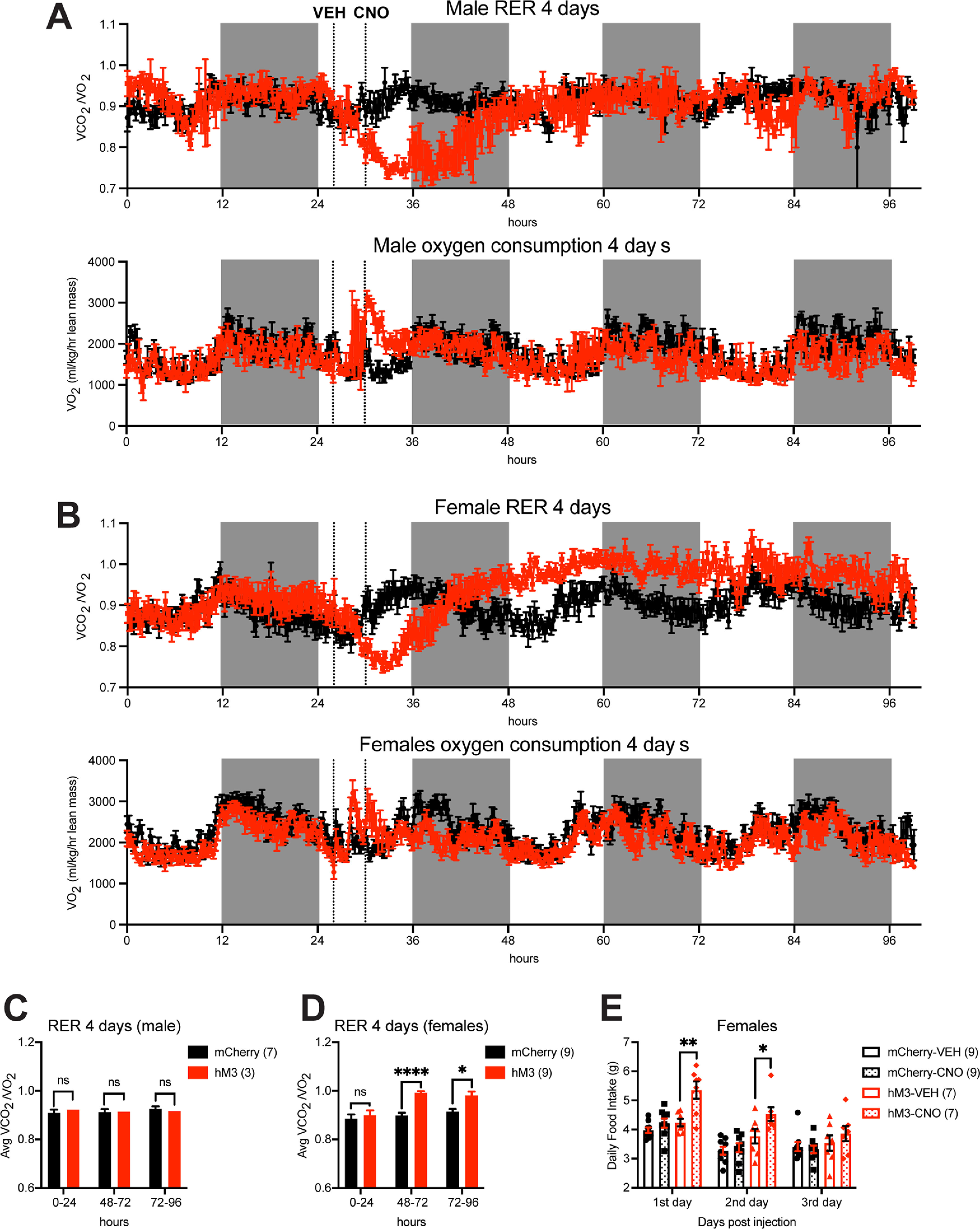
PVH^BDNF^ neuronal stimulation causes sustained increase in RER in female mice. ***A***, ***B***, RER (above) and oxygen consumption (VO_2_; below) in male and female *Bdnf^2A-Cre/+^* mice over the course of 4 d (1 d before and 3 d after VEH/CNO injection). Mice were injected bilaterally into the PVH with either AAV2-hSyn-DIO-mCherry [*n* = 7 male mice (RER), *n* = 3 male mice (VO_2_), and 9 female mice] or AAV2-hSyn-DIO-hM3D(Gq)-mCherry (*n* = 3 male mice and 9 female mice). RM two-way ANOVA over hours 48–96: males, *F*_(1,8)_ = 0.2182 and *p* = 0.6529 for RER, *F*_(1,4)_ = 0.9270 and *p* = 0.3902 for VO_2_; females, *F*_(1,16)_ = 39.58 and *p* < 0.0001 for RER, *F*_(1,16)_ = 3.025 and *p* = 0.1012 for VO_2_. ***C***, RER 24 h before and 24 and 48 h after CNO injection in male *Bdnf^2A-Cre/+^* mice. *n* = 7 mCherry mice and *n* = 3 hM3Dq mice. RM two-way ANOVA Bonferroni’s multiple comparisons test: ns = not significant. ***D***, RER 24 h before and 24 and 48 h after CNO injection in female *Bdnf^2A-Cre/+^* mice. *n* = 9 mCherry mice and 9 hM3Dq mice. RM two-way ANOVA Bonferroni’s multiple comparisons test: ns = not significant, **p < 0.01 and ****p < 0.0001. ***E***, Daily food intake of female *Bdnf^2A-Cre/+^* mice following VEH or CNO treatment. *n* = 9 mCherry mice and 7 hM3Dq mice. RM two-way ANOVA Bonferroni’s multiple comparisons test: *p < 0.05 and **p < 0.01. Data expressed as mean ± SEM.

Because RER in female hM3Dq mice appears to rebound over the control level within 24 h after CNO administration (Fig. 3*I*), we continued to measure RER for two additional days. We found that PVH^BDNF^ neuronal stimulation caused a sustained increase in RER for >2 d in hM3Dq females after a rapid decrease in RER (Fig. 4*B*,*D*). This delayed and long-lasting phenomenon shows sexual dimorphism and does not appear in male mice (Fig. 4*A*,*C*). In neither male nor female mice, CNO administration did not have any long-lasting effect on energy expenditure (Fig. 4*A*,*B*). One reason for the RER increase could be that following activation of PVH^BDNF^ neurons, female mice consume more regular chow that is rich in carbohydrates over the next few days. Female *Bdnf^2A-Cre/+^* mice indeed significantly increased food intake in the first 24-h period and to a lesser extent in the second 24-h period with no increase in the third 24-h period after PVH^BDNF^ stimulation compared with control mice (Fig. 4*E*); however, the increase in RER is sustained over the third day (Fig. 4*B*). Additionally, activation of PVH^BDNF^ neurons increased chow intake (Extended Data Fig. 4-1) without affecting RER (Fig. 4*A*) in male *Bdnf^2A-Cre/+^* mice in the second and third days. These results suggest that PVH^BDNF^ neuronal stimulation activates a compensatory mechanism against excess lipolysis or a regulatory mechanism that increases utilization of carbohydrates in female mice.

10.1523/ENEURO.0009-22.2022.f4-1 Extended Data Figure 4-1 Long-term effect of PVH^BDNF^ neuronal stimulation on food intake. Daily food intake of male *Bdnf^2A-Cre/+^* mice following VEH or CNO treatment. *n* = 11 mCherry mice and 5 hM3Dq mice. RM two-way ANOVA Bonferroni’s multiple comparisons test: *p < 0.05, **p < 0.01, and ****p < 0.0001. Data expressed as mean ± SEM. Download Figure 4-1, TIF file.

### Chronic PVH^BDNF^ neuronal stimulation leads to increased adiposity in female mice

**Figure 5.**
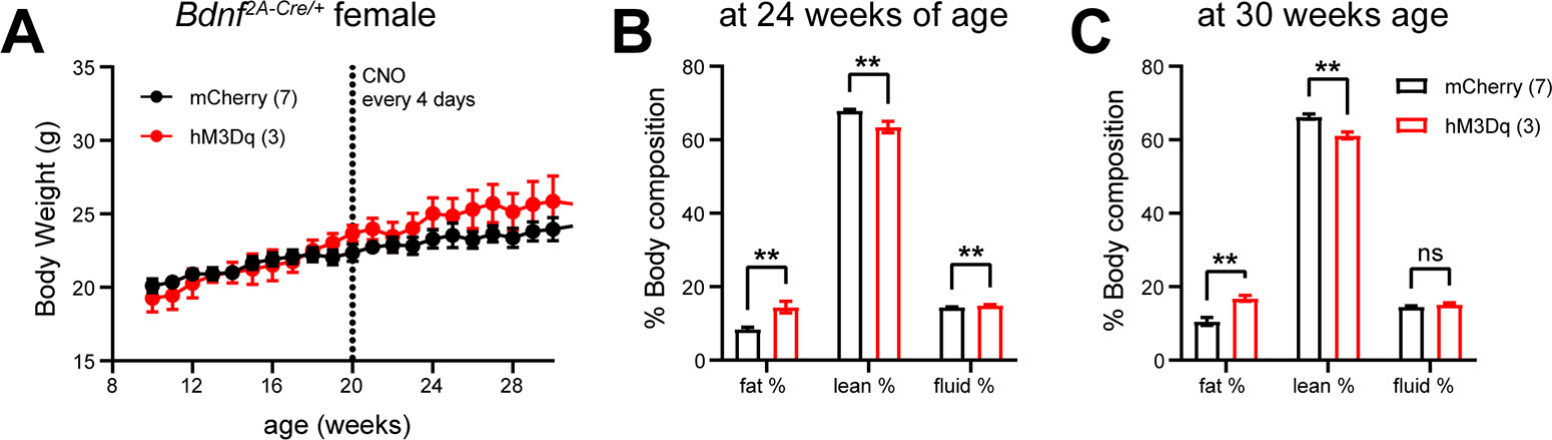
Chronic PVH^BDNF^ neuronal stimulation increases adiposity without altering body weight. ***A***, Body weight of female *Bdnf^2A-Cre/+^* mice injected into the PVH with either AAV2-hSyn-DIO-mCherry (mCherry mice, *n* = 7) or AAV2-hSyn-DIO-hM3D(Gq)-mCherry (hM3Dq mice, *n* = 3). Mice were treated with CNO (1 mg/kg) once every 4 d after they were 20 weeks old. RM two-way ANOVA: *F*_(1,8)_ = 0.6604 and *p* = 0.4399. ***B***, Body composition of mice at 24 weeks of age. Unpaired *t* test: **p < 0.01. ***C***, Body composition of mice at 30 weeks of age. Unpaired *t* test: ns = not significant and **p < 0.01. Data expressed as mean ± SEM.

The observation that a single chemogenetic activation of PVH^BDNF^ neurons increases RER for >2 d in female mice raises the possibility that multiple activations of these neurons may increase body weight and alter body composition in female mice. To test this hypothesis, we injected AAV expressing either mCherry or hM3Dq into the PVH of eight-week-old female *Bdnf^2A-Cre/+^* mice and then started to monitor body weight two weeks later under thermoneutral temperature. The two groups of mice had similar body weight from week 10 to week 20 (Fig. 5*A*), indicating that hM3Dq expression itself does not affect body weight. Afterwards, we chronically injected both groups of mice with CNO once every 4 d to stimulate PVH^BDNF^ neurons in the hM3Dq group for 10 weeks. The chronic CNO administration did not cause excess weight gain in hM3Dq mice (Fig. 5*A*). However, there was a significant increase in fat mass and decrease in lean mass in the hM3Dq group compared with the mCherry group after 4 (Fig. 5*B*) and 10 (Fig. 5*C*) weeks of CNO administration. Thus, these observations suggest that chronic PVH^BDNF^ neuronal stimulation increases adiposity in female mice.

## Discussion

It has been shown that deletion of the *Bdnf* gene in the PVH leads to hyperphagia, impaired adaptive thermogenesis, reduced energy expenditure, and perhaps reduced physical activity (An et al., 2015). In this study, we found that chemogenetic activation of PVH^BDNF^ neurons rapidly inhibited food intake, increased thermogenesis, elevated physical activity, and increased energy expenditure. These observations together demonstrate that PVH^BDNF^ neurons have a critical role in promoting negative energy balance by increasing satiety, adaptive thermogenesis, physical activity, and energy expenditure. Given that the majority of BDNF protein is released from neurons in an activity-dependent manner (Chen et al., 2006; An et al., 2008), these observations suggest that activation of PVH^BDNF^ neurons suppresses appetite and promotes energy expenditure by inducing BDNF release. Future studies are needed to determine whether distinct groups of PVH^BDNF^ neurons via single projections or one group of PVH^BDNF^ neurons via multiple collaterals is necessary to accomplish the regulation of appetite, physical activity, and adaptive thermogenesis. It is also important to understand how BDNF released from PVH^BDNF^ neurons modulates activities of these neural circuits to achieve negative energy balance.

Like BDNF, TrkB is expressed in the PVH and is critical for the control of energy balance. Deletion of the TrkB-coding *Ntrk2* gene in the adult PVH results in hyperphagia and severe obesity without affecting energy balance (An et al., 2020). Thus, it is unlikely that BDNF expressed in the PVH acts on PVH^TrkB^ neurons to regulate adaptive thermogenesis and physical activity. In fact, previous studies suggest that PVH^BDNF^ neurons release BDNF from their axonal terminals in the spinal cord to act on TrkB in sympathetic preganglionic neurons to promote adaptive thermogenesis (An et al., 2015; Wang et al., 2020). As activation of either PVH^BDNF^ or PVH^TrkB^ neurons strongly inhibits food intake (An et al., 2020), it is possible that BDNF expressed in the PVH regulates appetite through PVH^TrkB^ neurons. In this case, BDNF should mainly activate TrkB through a paracrine mode, given that there is a limited overlap between BDNF and TrkB expression in the PVH (An et al., 2015). However, we do not rule out the possibility that PVH^BDNF^ and PVH^TrkB^ neurons form distinct neural circuits and project to other brain regions to suppress appetite. These questions can be addressed in future studies using projection-specific gene deletion and projection-specific neuronal activation (An et al., 2020; Houtz et al., 2021).

As adaptive thermogenesis in brown adipocytes uses fatty acids as energy sources (Harms and Seale, 2013), increased adaptive thermogenesis is accompanied by elevated oxidation of fatty acids, leading to RER reduction. A recent study in the dorsomedial hypothalamus (DMH) reports that RER does not always correlate with changes in adaptive thermogenesis or energy expenditure when distinct groups of DMH^TrkB^ neurons are activated (Houtz et al., 2021), suggesting that RER is not only affected by adaptive thermogenesis but also subject to direct neuronal regulation. The results presented here further support this inference. We found that RER reduction lasts much longer than energy expenditure increase after PVH^BDNF^ neurons are activated. Thus, an increase in adaptive thermogenesis and/or physical activity, which would elevate energy expenditure, cannot be the only reason for RER reduction. It is of interest to identify the PVH^BDNF^ neurons that regulate fat metabolism.

It is a surprise that activation of PVH^BDNF^ neurons with a single dose of CNO causes a multiday RER increase in female (but not male) mice following an immediate and hours-long RER decrease. This could be a compensatory response to increased oxidation of fatty acids or negative energy balance following activation of PVH^BDNF^ neurons. Alternatively, the long-lasting RER increase is a result of activation of a specific group of PVH^BDNF^ neurons in female mice that modestly but persistently promotes utilization of carbohydrates as fuel. The latter hypothesis is consistent with our observation that the immediate RER reduction is shorter in females than in males following activation of PVH^BDNF^ neurons.

This overcompensatory response seen in females could be because of the influence of estrogen on BDNF/TrkB signaling in the brain and on energy homeostasis. Estrogen receptors colocalize to some neuronal populations that express BDNF and TrkB to regulate BDNF/TrkB expression and distribution, and this relationship has been shown to play a role in neurodegenerative diseases such as Parkinson’s and Alzheimer’s disease (Sohrabji and Lewis, 2006). Estrogen receptors (ERα subtype) are also expressed in the PVH and are responsible for increasing thermogenesis and energy expenditure (Liu and Shi, 2015). Furthermore, BDNF levels in plasma are positively correlated to estrogen levels in plasma. Thus, estrogen could account for the difference between males and females in BDNF expression and BDNF circulating throughout the brain. As estrogen receptors are expressed in the PVH, they could have the same effect on BDNF and TrkB signaling in the PVH. Hence, possible cross talk between estrogen signaling and this neurotrophin system could trigger an overcompensatory increase in energy balance in response to PVH^BDNF^ neuronal activation by stimulating negative feedback loops where estrogen is involved, thus implicating female mice as opposed to male mice.

Our chronic CNO administration experiment indicates that the delayed and long-lasting RER increase can lead to increased adiposity. Studies report that conscious efforts toward achieving weight loss through bariatric surgery or restricted food intake and extreme exercise regimens have been followed by long-term weight gain because of metabolic adaptation in the form of suppressed basal metabolic rate (Knuth et al., 2014; Fothergill et al., 2016). Our surprising finding on RER prompts us to speculate that increased utilization of carbohydrates is part of metabolic adaptation which makes it difficult to achieve long-lasting weight loss. It is important to understand how activation of PVH^BDNF^ neurons induces a delayed and long-lasting RER increase in female but not male mice in future studies.

## Synthesis

Reviewing Editor: Francisca Bronfman, Universidad Andrés Bello Decisions are customarily a result of the Reviewing Editor and the peer reviewers coming together and discussing their recommendations until a consensus is reached. When revisions are invited, a fact-based synthesis statement explaining their decision and outlining what is needed to prepare a revision will be listed below. The following reviewer(s) agreed to reveal their identity: Kate O’Neill, Esmeralda Fonseca.

In this work, the authors expand the field of neurotrophic factor control of energy balance by employing chemogenetic approaches to reveal both acute and long-term effects of brain-derived neurotrophic factor (BDNF) gene activation in paraventricular neurons (PVH). The authors’ motivation for this study is that deletion of the BDNF gene in PVH neurons contributes to obesity; thus, they chose to selectively and chemogenetically activate the BDNF gene in PVH neurons to determine whether the opposite effect (weight loss) occurs. This study has important implications for the treatment of obesity in humans.

Both reviewers recommend the study for publication but suggest revisions to the manuscript, as detailed below.

Major concerns:

-It is unclear whether both feeding protocols were conducted in the dark cycle. Reviewers

understood that the nocturnal food intake protocol was done during the dark

phase, but the fasting-induced feeding protocol during the light phase. If this is

the case, the decrease in the fasting-feeding group -which is already attenuated

compared to the nocturnal feeding- might be a product of the low amount of food

that mice consume during the light phase. Could the authors clarify this point? it would be advisable that both feeding protocols be performed under the dark cycle. The latter would preserve both feeding groups under similar circadian conditions while modifying their food deprivation state and would allow exploring if the anorexigenic effect induced by BDNF-PVH

neurons chemoactivation overrides hunger in a phase where the mice are naturally more active and willing to consume food.

-Could the authors comment on, why there are differences between the female control groups in the fasting-induced feeding protocol? Could this be related to an uncontrol circadian effect

as proposed above? Suppose the upcoming results keep showing these control

group differences only in the female group. In that case, the authors should include a couple

of lines in the discussion where they suggest alternative explanations for these results.

-Related to the Calculation of RER in Figure 4C-D. Why RER is averaged over 6 hours only (0-6 hours, 48-54 hours, and 72-78 hours)? Since the authors have continuous data, it should be averaged from [0-24), [48-72), and [72-96) to allow for proper comparison between these 24 hour periods.

Minor concerns:

-In the animal studies section (line 71), please include the total number of animals

used in the study.

-In the method section where you described your exclusion criterion, include how

many mice you removed from the study following each corresponding criterion.

-Physiological measurement section (lines 112-117): in the nocturnal food

protocol, the subjects were injected 20 minutes before having food access; in the

fasting-induced protocol, they were injected 30 minutes before. If this is a typo

mistake, please correct it; if not, I highly recommend giving the same time of

administration time for all groups.

-In lines 115-116: “food was removed the night before.” Could you please specify

how long lasted the food deprivation (i.e., 12 hours)?

-In line 116: include whether 9 am corresponds to the start or “x” hours after the

light or dark phase began.

-In line 192, the authors claim that “CNO administration produced much longer effects on RER... than on locomotor activity and oxygen consumption”. Was this tested statistically, and if so, were the findings significant? If statistical tests were not performed, then it is suggested softening this statement to the following: “CNO administration qualitatively appears to result in longer effects on RER... than on locomotor activity and oxygen consumption.”

-In line 208, the authors write, “...increased food intake in the first 24 hours and to a small extent in the second 24 hours but not in the 3rd day post...”. I would recommend changing this to read, “...significantly increased food intake in the first 24 hours period and to a lesser extent in the second 24 hours period with no increase in the third 24 hours period...”.

-Figure 3: Parts D-F and J-L should be labeled as “average” on the y axes. Same for Figure 4 C and D.

-Figure 4A-D: what do the dashed lines represent? It is assumed that the first line is vehicle administration and the second line is CNO administration. This should be labeled in the figure itself and included within the figure legend.

-Figure 4E and Extended Figure 2: the error bars are hard to see. I suggest either changing the color of the error bars or of the individual data points.

-The statistical tests used - repeated measures two-way ANOVA (followed by Bonferroni’s post-test) and t-tests seem appropriate. It is recommended that the authors change their unpaired (Student’s) t-tests in Figure 3 (specifically D, E, J, and K) to a paired t-test. Since the samples are not independent (same animals at different times), paired t-tests would be more appropriate.

Grammatical suggestions (not an exhaustive list, only what I noticed):

-line 168: suggest changing “activities of” to “activation of"

-line 204: suggest changing “had” to “did not have"

-line 243: suggest changing “as” to “is” and suggest changing “on” to “of"

-line 244: typo “as.”

-line 256: suggest changing “intrigue” to “interest"

For the Discussion section:

-Please consider discussing how TrkB may be involved in the processes addressed in the study (suggest adding this between the 1st and 2nd paragraph).

-A similar question with a different approach has already been addressed

(An et al., 2020). Please consider citing this work and highlight (in

the Discussion section) your approach differences and the gaining points -

that only by using these approaches- can be gained.

-Please consider adding a couple of lines with a proposed mechanism (communication within

the area (Zhou et al., 2020) or with other regions (Ann et al., 2020). Through which the activation of BDNF-PVH cells induces negative energy balance and weight loss.

-Please consider adding a couple of lines with a proposed mechanism by which there is an

over compensatory response in females but not males.

## Author Response

Point-by-point response to comments

We thank the reviewers and the editor for finding our study valuable, making constructive suggestions, and recommending the manuscript for publication after revision.

Major concerns:

It is unclear whether both feeding protocols were conducted in the dark cycle. Reviewers understood that the nocturnal food intake protocol was done during the dark phase, but (was) the fasting-induced feeding protocol (done) during the light phase? If this is the case, the decrease in the fasting-feeding group - which is already attenuated compared to the nocturnal feeding - might be a product of the low amount of food that mice consume during the light phase. Could the authors clarify this point?

Yes, the fasting-induced feeding protocol was done during the light phase. While the reviewers are correct that mice typically consume a lesser amount of food during the light phase compared to the dark phase in the non-fasted state, this would not be the case during an overnight fast. Previous studies as well as studies in our lab using this protocol have shown that after an overnight fast, mice are hyperphagic in the light phase. We add some details in Materials and Methods to clarify this issue.

It would be advisable that both feeding protocols be performed under the dark cycle. The latter would preserve both feeding groups under similar circadian conditions while modifying their food deprivation state and would allow exploring if the anorexigenic effect induced by BDNF-PVH neurons’ chemoactivation overrides hunger in a phase where the mice are naturally more active and willing to consume food.

Nocturnal feeding and fast-induced feeding are two feeding protocols that are frequently used in published studies. Nocturnal feeding is to examine hunger in a phase where mice are naturally more active and finish most of daily food intake without altering normal circadian rhythm. Circadian rhythm does not impair fast-induced feeding during the light cycle, as mice eat a significant amount of food after they are deprived of food overnight and given food at the early phase of the following light cycle (An et al., 2020, Nat Commun 11:1729). If we assess fast-induced hunger during the dark cycle, we cannot distinguish fast-induced hunger from the normal nocturnal hunger. Furthermore, food deprivation over one light cycle would not increase hunger much because mice do not eat much during the light cycle.

Could the authors comment on why there are differences between the female control groups in the fasting-induced feeding protocol? Could this be related to an uncontrol(led) circadian effect as proposed above? Suppose the upcoming results keep showing these control group differences only in the female group. In that case, the authors should include a couple of lines in the discussion where they suggest alternative explanations for these results.

While there are visible qualitative differences between the female control groups (mCherry-VEH, mCHerry-CNO, and hM3-VEH) in the fasting-induced feeding protocol, these differences are not statistically significant (Fig. 1F). The apparent but not significant difference between female mCherry-VEH and female hM3Dq-VEH is likely due to completely different sets of mice in these two groups. Importantly, there is not significant difference between mCherry-VEH and mCherry-CNO. We have added a sentence in Results to state this apparent difference and have added p values for comparisons between hM3Dq-VEH and hM3Dq-CNO to Figure 1 legend.

Related to the Calculation of RER in Figure 4C-D. Why RER is averaged over 6 hours only (0-6 hours, 48-54 hours, and 72-78 hours)? Since the authors have continuous data, it should be averaged from [0-24), [48-72), and [72-96) to allow for proper comparison between these 24 hour periods.

The reviewers make a valid point and thus we have changed the data to show averages in 24-hour periods in the revised manuscript.

Minor concerns:

In the animal studies section (line 71), please include the total number of animals used in the study. In the method section where you described your exclusion criterion, include how many mice you remove(d) from the study following each corresponding criterion.

The numbers are described in lines 95-97.

Physiological measurement section (lines 112-117): in the nocturnal food protocol, the subjects were injected 20 minutes before having food access; in the fasting-induced protocol, they were injected 30 minutes before. If this is a typo mistake, please correct it; if not, I highly recommend giving the same time of administration time for all groups.

This was a typo and has been corrected in the revised manuscript.

In lines 115-116: “food was removed the night before.” Could you please specify how long lasted the food deprivation (i.e., 12 hours)?

The food deprivation period has now been stated in the manuscript.

In line 116: include whether 9 am corresponds to the start or “x” hours after the light or dark phase began.

Done.

In line 192, the authors claim that “CNO administration produced much longer effects on RER... than on locomotor activity and oxygen consumption”. Was this tested statistically, and if so, were the findings significant? If statistical tests were not performed, then it is suggested softening this statement to the following: “CNO administration qualitatively appears to result in longer effects on RER... than on locomotor activity and oxygen consumption.”

We thank the reviewers for this suggestion, and the change has now been made in the manuscript.

In line 208, the authors write, “...increased food intake in the first 24 hours and to a small extent in the second 24 hours but not in the 3rd day post...”. I would recommend changing this to read, “...significantly increased food intake in the first 24 hours period and to a lesser extent in the second 24 hours period with no increase in the third 24 hours period...”.

This change has now been made in the manuscript.

Figure 3: Parts D-F and J-L should be labeled as “average” on the y axes. Same for Figure 4 C and D.

The y axes label has been changed to read ’average’ (Avg) preceding the measurement.

Figure 4A-D: what do the dashed lines represent? It is assumed that the first line is vehicle administration and the second line is CNO administration. This should be labeled in the figure itself and included within the figure legend.

This change has now been made in the manuscript for clarification.

Figure 4E and Extended Figure 2: the error bars are hard to see. I suggest either changing the color of the error bars or of the individual data points.

Suggested changes have been made to these figures.

The statistical tests used - repeated measures two-way ANOVA (followed by Bonferroni’s post-test) and t-tests seem appropriate. It is recommended that the authors change their unpaired (Student’s) t-tests in Figure 3 (specifically D, E, J, and K) to a paired t-test. Since the samples are not independent (same animals at different times), paired t-tests would be more appropriate.

We thank the reviewers for this recommendation and this change has now been made in the manuscript.

Grammatical suggestions (not an exhaustive list, only what I noticed):

line 168: suggest changing “activities of” to “activation of"

line 204: suggest changing “had” to “did not have"

line 243: suggest changing “as” to “is” and suggest changing “on” to “of"

line 244: typo “as.”

line 256: suggest changing “intrigue” to “interest"

Thank you for these suggestions. These changes have been incorporated into the manuscript and we have also carefully read through of the manuscript to correct any additional grammatical errors.

For the Discussion section:

Please consider discussing how TrkB may be involved in the processes addressed in the study (suggest adding this between the 1st and 2nd paragraph). A similar question with a different approach has already been addressed (An et al., 2020). Please consider citing this work and highlight (in the Discussion section) your approach differences and the gaining points - that only by using these approaches- can (these conclusions) be gained.

This suggestion has been incorporated into the manuscript.

Please consider adding a couple of lines with a proposed mechanism [communication within the area (Zhou et al., 2020) or with other regions (Ann et al., 2020)] through which the activation of BDNF-PVH cells induces negative energy balance and weight loss.

This suggestion has been incorporated into the manuscript.

Please consider adding a couple of lines with a proposed mechanism by which there is an over compensatory response in females but not males.

This suggestion has been incorporated into the manuscript.
