# Supplementary figures and images for: Rapid and Lasting Effects of Activating BDNF-Expressing PVH Neurons on Energy Balance
Source: eNeuro. 2022 Apr 5;9(2):ENEURO.0009-22.2022. doi: 10.1523/ENEURO.0009-22.2022 (PMC8994543; doi:10.1523/ENEURO.0009-22.2022)

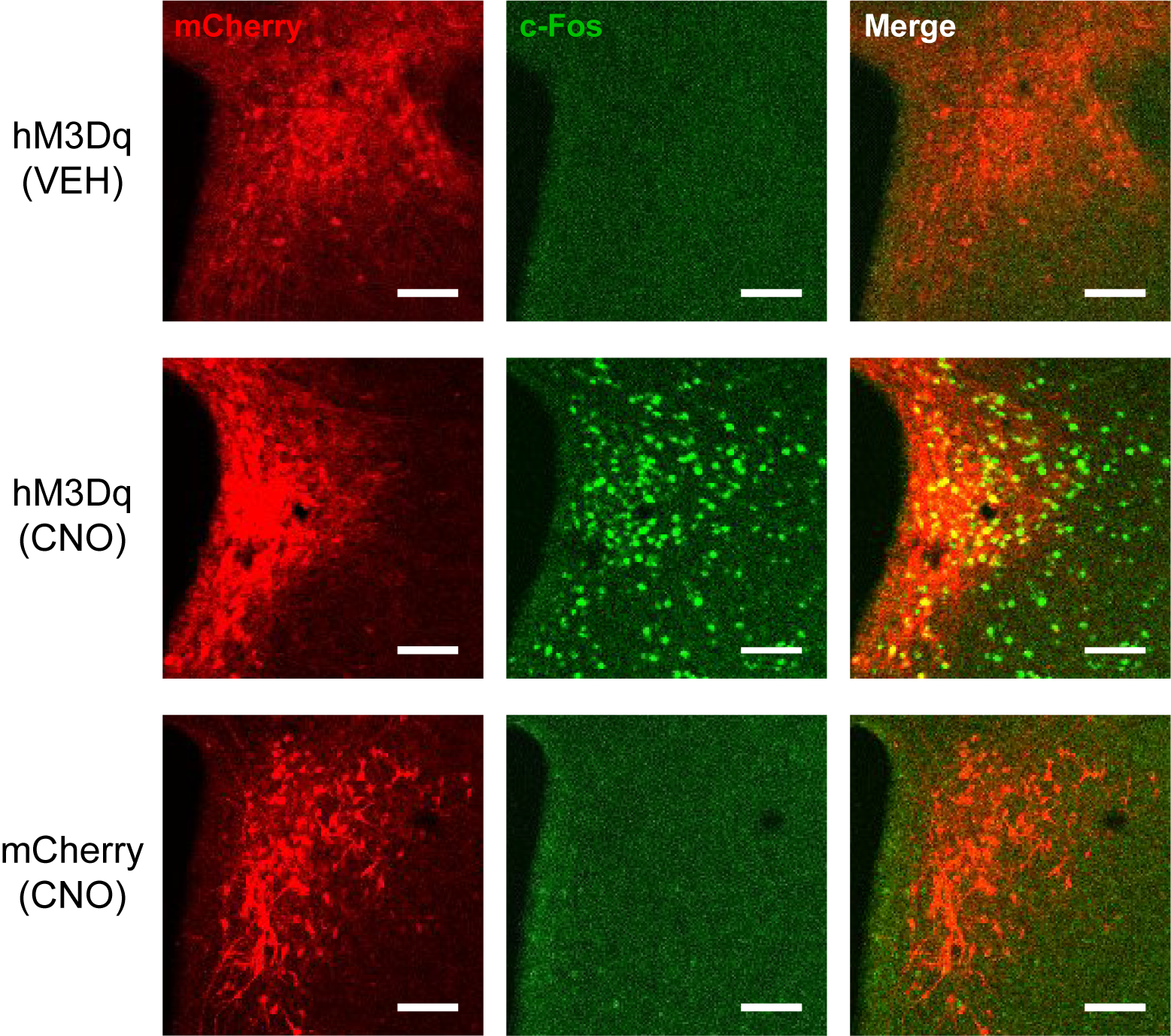

Supplement: Extended Data Figure 1-1 — CNO administration induces Fos expression in hM3Dq mice. Representative images of Bdnf2A-Cre/+ mice injected into the PVH with AAV2-hSyn-DIO-mCherry or AAV2-hSyn-DIO-hM3D(Gq)-mCherry (hM3Dq). Mice were treated with vehicle (VEH) or CNO (1 mg/kg) 1 h prior to euthanization. Top row, Mouse injected with AAV2-hSyn-DIO-hM3D(Gq)-mCherry (hM3Dq) and treated with VEH. Middle row, Mouse injected with AAV2-hSyn-DIO-hM3D(Gq)-mCherry (hM3Dq) and treated with CNO. Bottom row, Mouse injected with AAV2-hSyn-DIO-mCherry (mCherry) and treated with CNO. Scale bar: 100 μm. Download Figure 1-1, TIF file. [file enu-eN-NWR-0009-22-s02.tif]

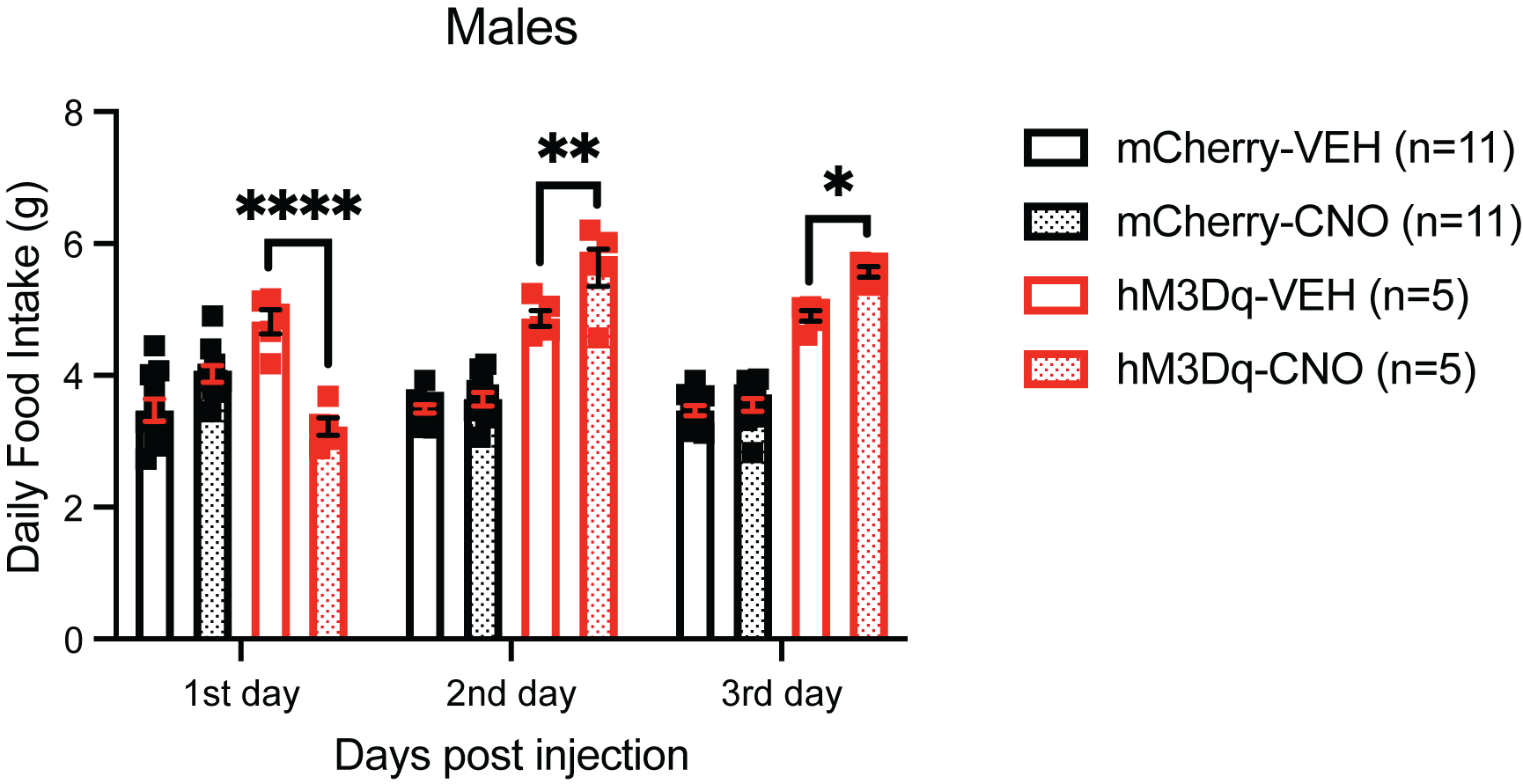

Supplement: Extended Data Figure 4-1 — Long-term effect of PVHBDNF neuronal stimulation on food intake. Daily food intake of male Bdnf2A-Cre/+ mice following VEH or CNO treatment. n = 11 mCherry mice and 5 hM3Dq mice. RM two-way ANOVA Bonferroni’s multiple comparisons test: *p < 0.05, **p < 0.01, and ****p < 0.0001. Data expressed as mean ± SEM. Download Figure 4-1, TIF file. [file enu-eN-NWR-0009-22-s03.tif]
